# Novel Capsid-Specific Single-Domain Antibodies with Broad Foot-and-Mouth Disease Strain Recognition Reveal Differences in Antigenicity of Virions, Empty Capsids, and Virus-Like Particles

**DOI:** 10.3390/vaccines9060620

**Published:** 2021-06-08

**Authors:** Haozhou Li, Aldo Dekker, Shiqi Sun, Alison Burman, Jeroen Kortekaas, Michiel M. Harmsen

**Affiliations:** 1 Laboratory of Virology, Wageningen University and Research, 6708 PB Wageningen, The Netherlands; Haozhou.li@wur.nl (H.L.); jeroen.kortekaas@wur.nl (J.K.); 2 State Key Laboratory of Veterinary Etiological Biology and National Foot and Mouth Disease Reference Laboratory, Lanzhou Veterinary Research Institute, Chinese Academy of Agricultural Sciences, Lanzhou 730046, China; sunshiqi@caas.cn; 3 Wageningen Bioveterinary Research, P.O. Box 65, 8200 AB Lelystad, The Netherlands; Aldo.Dekker@wur.nl; 4 The Pirbright Institute, Ash Road, Pirbright Surrey GU24 0NF, UK; alison.burman@pirbright.ac.uk

**Keywords:** foot-and-mouth disease, virus-like particles, vaccine, nanobody, single-domain antibody, immunogenicity

## Abstract

Foot-and-mouth disease (FMD) vaccine efficacy is mainly determined by the content of intact virions (146S) and empty capsids (75S). Both particles may dissociate into 12S subunits upon vaccine manufacturing, formulation, and storage, reducing vaccine potency. We report the isolation of capsid-specific llama single-domain antibodies (VHHs) with broad strain recognition that can be used to quantify intact capsids in FMD vaccines by double antibody sandwich (DAS) ELISA. One capsid-specific VHH displayed remarkably broad strain reactivity, recognizing 14 strains representing the 13 most important lineages of serotype A, and two VHHs cross-reacted with other serotypes. We additionally show that the newly isolated VHHs, as well as previously characterized VHHs, can be used to identify antigenic differences between authentic 146S and 75S capsids, as well as corresponding genetically engineered virus-like particles (VLPs). Our work underscores that VHHs are excellent tools for monitoring the quantity and stability of intact capsids during vaccine manufacturing, formulation, and storage, and additionally shows that VHHs can be used to predict the native-like structure of VLPs.

## 1. Introduction

Foot-and-mouth disease (FMD) affects cloven-hoofed animals, such as cattle, pigs, and sheep. Its ability to spread rapidly across geographic areas and cause large epizootics poses a continuous threat to the livestock industry, especially in developing countries. FMD is caused by FMD virus (FMDV), an aphthovirus of the family *Picornaviridae*. The RNA genome of FMDV is surrounded by a capsid assembled from 60 protomers, each comprising one copy of the four capsid proteins VP1, VP2, VP3, and VP4. The latter protein is located on the inside of the particle. The capsid proteins adopt a T = 3 pseudo-icosahedral structure with VP1 located close to the fivefold axes of symmetry and VP2 and VP3 alternating around the threefold axes [1]. Five protomers assemble into a pentamer (12S), and 12 pentamers assemble into an integrated FMDV capsid. 

FMDV exists as seven serotypes: A, O, C, Asia1, SAT1, SAT2, and SAT3. The most prevalent serotypes in the field are O and A, followed by SAT2 and Asia1. FMD can be controlled effectively through vaccination. However, due to antigenic variation, vaccines do not elicit cross-protection. Conventional FMD vaccines are mostly multivalent and are based on chemically inactivated FMDV strains formulated with an adjuvant. Apart from these classical vaccines, next-generation vaccines based on FMD virus-like particles (VLPs) are being developed that mimic the native structure of the FMDV particle, produced with mammalian-, insect- or bacterial-cell expression systems [2,3,4,5]. 

FMDV virions sediment at 146S in sucrose density gradients (SDG), whereas natural empty capsids lack the viral genome and VLPs sediment at 75S. Incubation of intact capsids at high temperatures or low pH leads to irreversible dissociation into 12S subunits, resulting in a strongly reduced immunogenicity [6,7,8]. Consequently, the 146S content of conventional vaccines and 75S content of VLP vaccines determines vaccine quality and should thus be monitored closely during vaccine manufacturing, formulation, and storage [9,10,11,12].

Several methods have been developed to quantify FMDV 146S antigens for use in vaccines. SDG fractionation and 146S quantification by short-wavelength (254–260 nm) UV absorbance to measure RNA content is the traditional and most commonly used method. Such SDG is not suitable for quantification of VLPs and natural 75S capsids since these are not detected by 254–260 nm absorbance. It is also not suitable for high throughput applications and relies on delicate manual operation, making it difficult to standardize [13]. Novel methods, including size-exclusion chromatography and high-performance liquid chromatography [14,15,16], are easier to perform and standardize. Disadvantages of these methods include low sensitivity, limited sample throughput, and inability to quantify different vaccine strains in multivalent vaccines. Double antibody sandwich (DAS) ELISAs using monoclonal antibodies (mAbs) or polyclonal antibodies (pAbs) were also developed for FMDV antigen quantification [17,18,19]. These assays are more sensitive and scalable than the aforementioned methods but are generally not 146S specific. Two DAS-ELISAs were previously developed that can be used to quantify 146S content of serotype A and O strains by making use of 146S-specific mAbs [20,21]. The development of 146S-specific mAbs that can be used in DAS-ELISAs is, however, complicated due to the high amino acid variation within FMDV antigenic sites [22], common epitopes shared by 146S and 12S particles, and the long and complicated screening procedure of mAbs. As an alternative to mAbs, we make use of recombinant single-domain antibodies derived from heavy-chain-only antibodies of llamas, known as VHHs. The small size of VHHs enables the use of phage-display libraries for selection and facilitates large-scale manufacturing in bacteria and yeast. We previously discovered that a panel of 24 earlier isolated VHHs against FMDV strain O1/Manisa/TUR/69 [23] contained a 146S-specific VHH: clone M170 [9]. Further 146S-specific VHHs were isolated against strains Asia1/Shamir/ISR/89 and SAT2/SAU/2/00 [24]. These were used in DAS-ELISA to measure the stability of the FMDV antigens before and after oil emulsification and the stabilizing effects of excipients [9].

The primary objective of the present work was to isolate 146S-specific VHHs against FMDV serotype A, which is antigenically the most heterogeneous serotype [25,26]. In addition, we aimed to broaden our panel of 146S-specific VHHs for serotypes O and Asia1. We isolated 1 novel 146S-specific VHH for strain Asia1/Shamir/ISR/89 and 12 novel 146S-specific VHHs against serotype A strains. Four of these were broadly reactive with serotype A strains. Surprisingly, some serotype A 146S-specific VHHs did not bind natural 75S particles. We, therefore, expanded our study to look for differences in the antigenicity of 146S particles and various VLPs of strains from different serotypes using both novel and previously isolated 146S-specific VHHs. Wild-type (wt) and stabilized mutant VLPs showed differences in antigenicity for serotypes O, A, and SAT2. Thus, 146S-specific VHHs can be used not only for quantification but also for the prediction of the native-like structure of VLPs.

## 2. Materials and Methods

### 2.1. VHHs and FMDV Antigens

Earlier isolated VHHs used are shown in Table 1.

The FMDV strains A/IRN/2/87, A/TUR/20/2006, A/MAU/1/2006, A/ETH/4/2007, A/KEN/12/2005, A/ETH/13/2005, A/SUD/2/84, A/ERI/2/98, and A10/HOL/1/42, were propagated at Wageningen Bioveterinary Research [27]. A/TUR/14/98, A22/IRQ/24/64, A24/Cruzeiro/BRA/55, Asia1/Shamir/ISR/89, C1/Detmold/FRG/60, O1/BFS 1860/UK/67, O1/Manisa/TUR/69, and O/TAW/3/97 originated from the virus production facilities in Lelystad, as earlier described [24]. Briefly, FMDV was amplified in BHK- 21 cells grown in suspension in industrial-size bioreactors or 850-cm^2^ roller bottles. FMDV present in the clarified supernatant was inactivated with 10 mM binary ethylenimine and concentrated using polyethylene glycol-6000 precipitation, resulting in crude antigen. The FMDV strains AF72, A/HuBWH/CHA/2009, A/GDMM/CHA/2013, and O/BY/CHA/2010 were obtained from the OIE/National Foot-and-Mouth Disease Reference Laboratory of the Lanzhou Veterinary Research Institute, Chinese Academy of Agricultural Sciences, Lanzhou, China. Antigens were produced as earlier described [28]. Briefly, FMDV was amplified in BHK-21 monolayers until cytopathic effect reached 90%. Culture supernatant was harvested and FMDV was inactivated as described above. An ultrafiltration system (Merck Millipore, Germany) was used to concentrate the FMDV preparations.

VLPs of FMDV strain A/GDMM/CHA/2013 were generated by expression in *E. coli* using purified small ubiquitin-like modifier (SUMO) protein-tagged recombinant proteins and self-assembly in vitro as previously described [2]. The protein content was determined using a Bradford protein assay kit in accordance with the manufacturer’s instructions (ThermoFisher Scientific, Rockford, IL, USA).

VLPs of strains O1/Manisa/TUR/69, A22/IRQ/24/64, Asia1/Shamir/ISR/89, and SAT2/ZIM/7/83 were produced by a vaccinia virus expression system at The Pirbright Institute. They were produced as both wt and either one or more stabilizing mutations at VP2 residue 93 from a serine (O1/Manisa/TUR/69, Asia1/Shamir/ISR/89, and SAT2/ZIM/7/83) or a histidine (A22/IRQ/24/64) to either cysteine (93C), phenylalanine (93F) or tyrosine (93Y), as described earlier, for serotype O, A and SAT2 [3,5]. The VLPs of serotype Asia1 were produced in a similar manner. Generally, the VLPs were named according to the wt strains and their mutation at VP2 residue 93 as wt, 93C, 93F, or 93Y. Note that the P1 coding region of the O1/Manisa/TUR/69 strain used for VLP generation (isolate 87; AY593823) differs from the authentic virus strain used in this study (FN594747) at 6 amino acid positions, and the A22/IRQ strain used for VLP generation (isolate 95; AY593762) differs from the authentic virus strain used (MN447655) at 5 amino acid positions. 

FMDV particles (inactivated authentic virus) were fractionated using 10–40% sucrose density gradients (SDG) and centrifuged for 2 h at 10 °C and 200,000× *g*. The gradients were fractionated into twenty 0.60 mL aliquots, and the absorbance at 254 nm was determined to identify the 146S peak. The 146S concentration in μg/mL was then calculated by multiplying the absorbance at 254 nm by 126.7 [29]. We previously described the treatment of authentic particle preparations for 1 h at 56 °C to prepare 12S particles [24]. The concentration of 12S particles was derived from the 146S concentration of the sample from which it was prepared, assuming complete conversion of 146S into 12S particles.

### 2.2. Llama Immunization and Phage Library Construction

Immunizations of llamas with various FMDV strains are summarized in Table S1. The immunizations of llamas 3049, 6058, 6666, and 7212 have been described previously [23,24]. Llama 9245 was intramuscularly immunized three times with a three-week interval with 50 μg 146S of A22/IRQ/24/64 and 50 μg 146S of O1/Manisa/TUR/69 each time, whereas llama 9246 received 50 μg 146S of A/TUR/14/98 according to the same schedule. In the fourth and seventh weeks post-immunization, the VHH repertoire of individual llamas was amplified by RT-PCR from peripheral blood lymphocytes and separately inserted into phage display vector pRL144 as described earlier [23,24].

### 2.3. Phage Display Selection of FMDV Binding VHHs

Libraries consisting of at least 10^7^ unique clones were generated, and phages were rescued. Phage display selections were performed by two consecutive rounds of biopanning in polystyrene 96-well plates (Greiner, Solingen, Germany), using 100 µL/well for each incubation. SDG-purified 146S from strains A/TUR/14/98, A24/Cruzeiro/BRA/55, A22/IRQ/24/64, O/TAW/3/97, and Asia1/Shamir/ISR/89 was used for phage display selections. These FMDV authentic 146S particles were immobilized using a specific capturing VHH to prevent capsid denaturation due to passive adsorption during the biopanning procedures. Each FMDV 146S particle was used in two separate biopanning procedures using either the broadly reactive capturing VHH M8F or using one of the serotype/strain-specific VHHs M326F, M321F, M23F, or M98F (Table 1). The use of a serotype-specific VHH for the capture of 146S particles enabled the competition with 12S from O1/BFS/1860/UK/67 when selecting on serotype A or Asia1 strains or 12S from A/TUR/14/98 when selecting on O/TAW/3/97. In general, earlier isolated capturing VHHs were coated using concentrations of 0.1–1 μg/mL in 0.05 M carbonate/bicarbonate buffer, pH 9.6 (coating buffer), overnight at 4 °C. FMDV preparations (1 μg/mL) were separately incubated in PBS buffer containing 1% skim milk and 0.05% Tween-20 (PBSTM) for 45 min at RT. Controls included wells coated with VHH without subsequent incubation with FMDV authentic particles and uncoated wells incubated with 1 μg/mL FMDV authentic particles. Plates were then incubated with 1 × 10^9^ transducing units phages per well. When using a serotype-specific capturing VHH, phages were sometimes preincubated with 20 μg/mL 12S particles from another serotype. Bound phages were finally eluted by incubation with 1 mg/mL trypsin in PBS for 30 min at 37 °C and immediately transduced to *Escherichia coli* TG1 ((F′ traD36 proAB lacIqZ ΔM15) supE thi-1 Δ(lac-proAB) Δ(mcrB-hsdSM)5(rK− mK−)) cells. In each selection round, a phage ELISA was performed, simultaneously with phage display selection. For this purpose, a duplex plate containing similar concentrations and types of FMDV 146S particles and phage was incubated with a peroxidase-conjugated mAb against M13 phage instead of incubation with trypsin. The amount of bound 146S particle-specific phage was then measured by phage ELISA.

After the second round of panning, phages were transduced to *E. coli* TG1 cells, and individual colonies were picked. The expression of the VHH genes was induced by adding 1 mM isopropyl β-d-thiogalactopyranoside (IPTG). Soluble recombinant VHHs, directed to the periplasm, were tested for binding to FMDV authentic particles as described below in Section ELISAs.

### 2.4. Sequence Analysis

For sequencing the P1 region of the various FMDV strains, cDNA was synthesized using superscript II reverse transcriptase (ThermoFisher Scientific) and primer FMDV-116 (5′-GACATGTCCTCCTGCATCTG-3′). A 3.5 kB PCR fragment was generated using the Expand Long Template PCR system (Roche Applied Science, Mannheim, Germany) and primer FMDV-83 (5′-CCCCCCCCCCCCCCCCCCCCTAGGT-3′) in combination with either FMDV-116 or FMDV-115 (5′-GACATGTCCTCCTGCATCTGGTTGAT-3′). PCR products purified from agarose gel as well as individual VHHs were sequenced using both strands by fluorescent dideoxy DNA sequencing using an ABI Prism^®^ 3130 Genetic Analyzer and the BigDye Terminator V1.1 Cycle Sequencing kit (ThermoFisher Scientific). The SeqMan Pro program of the Lasergene suite was used to align and merge the sequence reads. The deduced VHH amino acid sequences were aligned according to the IMGT system [30] for alignment, numbering, and CDR definition of immunoglobulins. VHHs were classified into subfamilies as earlier defined [31]. VHHs were classified into CDR3 groups based on the CDR3 sequence, which is the most variable among VHHs [31]. Clones from the same CDR3 group have identical CDR3 length and at least 75% CDR3 sequence identity. Potential N-glycosylation sites were defined as Asn-X-Ser/Thr, where X represents any amino acid, except Pro.

### 2.5. Production of Selected VHHs in Yeast

VHHs fused to the natural llama heavy-chain antibody long hinge region, and a hexahistidine (his6) tag was produced in baker’s yeast using vector pRL188 and purified from culture supernatant using immobilized-metal affinity chromatography as described earlier [23]. VHHs produced in this manner were indicated by the suffix “F.” About 10% of the VHH amount produced in this manner was dimerized through the single cysteine present at the C-terminus of the VHH, immediately preceding the his6 tag. Both monomeric and dimeric VHHs are useful for functional immobilization of VHHs to polystyrene surfaces by passive adsorption [9,24]. Yeast-produced VHHs were biotinylated at a weight ratio of protein to biotin of 5 using amine-reactive sulfo-N-hydroxysuccinimide-LC-biotin (ThermoFisher Scientific).

VHHs were subjected to reducing SDS-PAGE using precast gels (Novex, San Diego, CA, USA), and stained using GelCode Blue reagent (ThermoFisher Scientific). VHHs were digested with endoglycosidase H (Roche Applied Science) according to the manufacturer’s instructions. 

### 2.6. ELISAs

Three different ELISA procedures were used. We first describe the basic ELISA procedure that was used in all three procedures. ELISAs were performed by coating high-binding polystyrene 96-well plates (Greiner) with 0.1–1 μg/mL of unlabeled VHH in 50 mM carbonate/bicarbonate buffer, pH 9.6 (coating buffer), overnight at 4 °C. Coating and subsequent incubations were performed using 100 µL per well. After washing, the coated plates were incubated with FMDV particles in ELISA buffer (1% skimmed milk; 0.05% Tween-20; 0.5 M NaCl; 2.7 mM KCl; 2.8 mM KH_2_PO_4_; 8.1 mM Na_2_HPO_4_; pH 7.4) or PBSTM for 45 min at RT. Plates were next incubated with either phage-displayed VHH, *E. coli*-produced VHH, or yeast-produced biotinylated VHH, and subsequently with a suitable specific peroxidase conjugate, using the same buffer as used for incubation with FMDV particles (ELISA buffer or PBSTM). The peroxidase conjugate was subsequently detected by staining with 3,3′,5,5′-tetramethylbenzidine. After stopping the reaction by the addition of 0.5 M H_2_SO_4_ (50 µL per well), the absorbance at 450 nm was measured using a Multiskan Ascent spectrophotometer (Thermo Labsystems, Helsinki, Finland). 

For phage ELISAs, plates were coated with low concentrations of VHH (0.1–1 μg/mL) and FMDV 146S particles (1 μg/mL) as described before [24]. Bound phages were detected by incubation with peroxidase-conjugated mAb against the M13 p8 coat protein (GE Healthcare, Little Chalfont, UK) in PBSTM. 

For ELISA analysis of *E. coli*-produced soluble VHH, plates were preferably coated with 1 μg/mL yeast-produced VHH and then subsequently incubated with 1 μg/mL FMDV authentic particles in ELISA buffer. Bound FMDV authentic particles were detected by incubation with 10-fold diluted *E. coli*-produced soluble VHH, which contains a myc-tag, and a peroxidase-conjugated mAb against the c-myc tag (clone 9E10; Roche Applied Science). Four ELISAs with different capturing VHH/FMDV authentic particles combinations were conducted to evaluate the 146S specificity of the *E. coli*-produced VHH, with two parallel comparisons done in each ELISA. One comparison used the M326F-, M321F-, M98F- or M23F-captured authentic particles versus M3F-captured 12S, and the other comparison used M8F for comparing authentic particles with 12S.

Plates were coated with 0.5 μg/mL of unlabeled yeast-produced VHH to determine FMDV particle concentrations by double antibody sandwich (DAS) ELISA. These plates were then incubated with serial 2-fold dilution series of FMDV particle preparations in ELISA buffer. Normally, standards of untreated FMDV particles were included in the ELISAs for the quantification of FMDV particles. Standards were serially 2-fold diluted using both 1 μg/mL and 0.66 μg/mL as starting concentrations. Plates were next incubated with 0.25 μg/mL of biotinylated VHH. The different DAS-ELISAs employed the same VHH for coating in unlabeled form and for detection in biotinylated form. Bound biotinylated VHH was detected with 1 μg/mL peroxidase-conjugated streptavidin (Jackson ImmunoResearch Laboratories Inc., West Grove, PA, USA). Absorbance data were evaluated using an Excel spreadsheet template (Microsoft Corporation, Redmond, WA, USA). A four-parameter logistic curve was fitted to absorbance and FMDV particle concentrations of standards by non-linear least squares using the Excel solver tool. The FMDV particle concentration in unknown samples was then determined by interpolation. For the determination of the binding of VHHs to particular FMDV particles, the particles were titrated in two or three-fold dilution series. The effective particle concentration (EC) required to reach an absorbance value of 1 was then interpolated after four-parameter logistic curve fitting of absorbance and particle concentrations. The LOD of ELISAs was measured by titrating FMDV particles in triplicate and interpolating the FMDV particle concentration required to reach the average absorbance value of background plus 3 times the standard deviation.

## 3. Results

### 3.1. Isolation of 146S-Specific VHHs

Phagemid libraries from four llamas previously immunized and two llamas immunized in this study (Table S1) were used for the selection of 146S-specific VHHs against three serotype A strains as well as Asia1/Shamir/ISR/98 and O/TAW/3/97. After two panning rounds, soluble VHH of 1260 individual clones was analyzed for 146S specificity in two separate ELISA setups using earlier isolated VHHs (Table 1) for antigen capture. We used either M8F or a serotype-specific VHH for the capture of 146S particles and either M8F or M3F for the capture of 12S particles. In total, 180 ELISA-positive clones were subsequently further analyzed by ELISA, using all five FMDV strains in both ELISA setups for each clone to confirm their 146S specificity and determine their serotype/strain specificity. From this analysis, 108 clones were selected for determining the VHH sequence primarily based on their specificity for 146S particles. However, some clones without 146S specificity were also selected based on their serotype or strain specificity, which was either broad or narrow, simply to retain a broad panel of VHHs. These 108 clones represented 67 unique clones which were named M642 to M717. They formed 31 complementarity-determining region 3 (CDR3) groups. 

In total, 26 novel VHHs belonging to 20 CDR3 groups were produced in yeast for further study (Table 2). They were selected primarily based on their 146S- and serotype/strain specificity. Isolation of 146S-specific VHHs specific for O/TAW/3/97, however, was unsuccessful. The selection criteria also included the presence of restriction sites relevant for subcloning and the absence of potential N-glycosylation sites since this could abrogate FMDV binding of the yeast-produced VHH when such sites are indeed N-glycosylated. Only clones M651F and M655F containing an N-glycosylation site were included (Table 2) because they are single representants of a CDR3 group. VHH M658F belongs to the same CDR3 group as M332F, which was earlier isolated [24], whereas all 19 further CDR3 groups were novel. None of the isolated VHHs belonged to the VHH conventional-like subfamily or subfamily 3 that contains an additional disulfide bond (Table 2).

**Figure 1:**
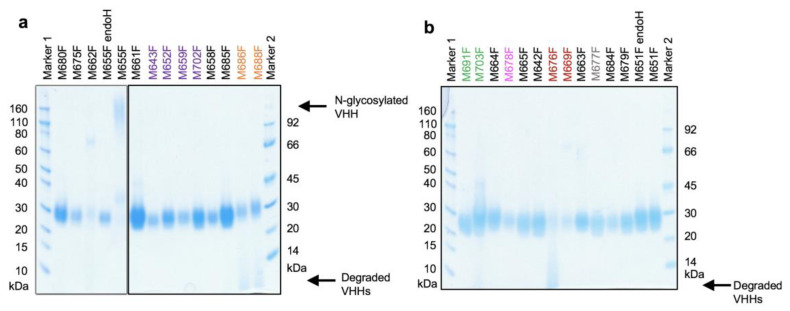
Analysis of yeast-produced VHHs by reducing SDS-PAGE. VHHs M651F and M655F, which contain potential N-glycosylation sites, were treated with endoglycosidase H (endoH). VHHs were analyzed on two gels (panels (**a**,**b**)) and are arranged according to their CDR3 group. A single lane left of M661F was removed from panel a. Newly isolated VHHs of the same CDR3 group are indicated using the same color. The position of N-glycosylated M655F and degradation products of VHHs are indicated at the right. The molecular weight of markers used is indicated.

All 26 yeast-produced VHHs migrated as diffuse bands in SDS-PAGE, suggestive of O-glycosylation, with a molecular weight of about 24 kDa (Figure 1), as expected for a VHH containing an O-glycosylated llama heavy-chain antibody long hinge region and his6 tag. Of the two clones (M651F and M655F) having a potential N-glycosylation site, only M655F was found to contain an N-linked glycan, which was removed by enzyme treatment. All subsequent studies were done using deglycosylated M655F. The VHHs M676F, M686F, M688F, and M703F were partially degraded.

### 3.2. Particle and Strain/Serotype Specificity of VHHs

**Figure 2:**
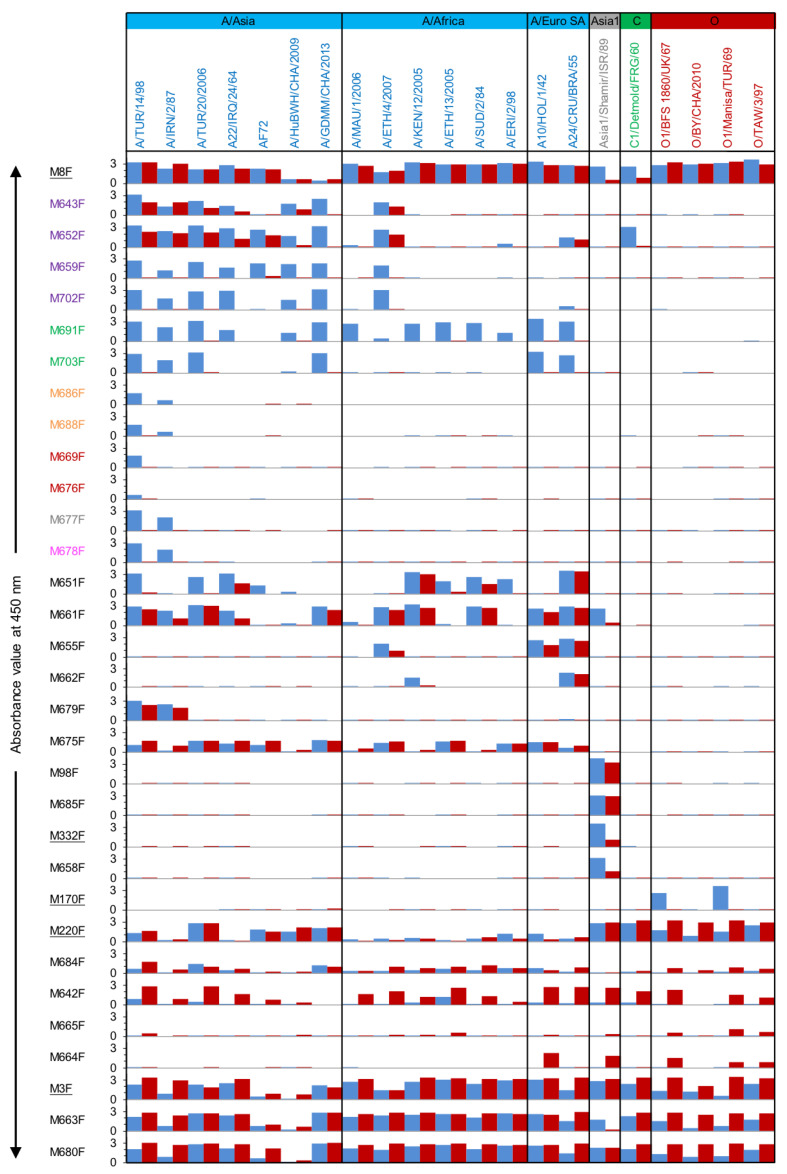
FMDV particle- and strain-specificity of VHHs. DAS-ELISAs were performed using a matrix of 32 VHHs and 21 FMDV strains representing four serotypes. Each strain was used in ELISA at 1 µg/mL 146S either as untreated authentic particles (blue) or after conversion into 12S by heating (red). The absorbance value at 450 nm after subtraction of the background absorbance without FMDV particles is shown. The 12 VHHs of the 6 CDR3 groups comprising the 10 VHHs that bind specifically to 146S of serotype A strains are color-coded by their CDR3 group. The control VHHs isolated earlier are underlined. The VHHs are arranged from top to bottom according to their CDR3 group and their particle and strain specificity. Broadly reactive control VHH M8F, however, is placed on top to show all FMDV particle preparations contain immunoreactive material. FMDV strains are arranged from left to right according to their phylogenetic relationship (Figure 3).

The yeast-produced VHHs were analyzed for FMDV particle specificity in a DAS-ELISA setup using the same VHH for coating in an unlabeled form and detection of FMDV particles in biotinylated form. Furthermore, to determine the FMDV strain/serotype specificity of the novel VHHs, 21 vaccine strains were used as FMDV particles in this DAS-ELISA (Figure 2 and Figure S1). Three broadly reacting VHHs, M3F (recognizing 12S of all 21 strains), M8F, and M220F (recognizing 146S and 12S of all 21 strains), which recognize independent antigenic sites, were used to verify the presence of antigenic particles from the various strains. Most of this experiment was performed in the Netherlands, with additional analyses performed in China, as indicated in Figure S1. The same batch of A/TUR/14/98 was used in both experiments/countries to compare the two ELISAs, and the results were in agreement. However, background values without FMDV particles differed between the experiments for a number of VHHs. We, therefore, used background-subtracted values for graphical presentation in Figure 2.

VHHs from the same CDR3 group generally show similar particle- and strain-specificity. Overall, for serotype A, VHHs M659F, M702F, M691F, M703F, M686F, M688F, M669F, M676F, M677F, M678F, which encompass six CDR3 groups, bound to untreated particles of strain A/TUR/14/98 and a variable number of additional serotype A strains while not binding to heated (12S) particles of any of these strains (Figure 2). These 10 consistently 146S-specific VHHs were selected for further work. However, two VHHs with no specificity for 146S of most strains, M643F and M652F, were also selected since they belonged to the same CDR3 group as two 146S-specific VHHs, M659F and M702F. These 12 VHHs are color-coded by their six CDR3 groups, purple, green, orange, red, gray, and magenta, respectively (Table 2). This color-coding is also used in subsequent Figures and Tables to highlight these important VHHs. Of note, VHH M658F showed similar 146S specificity to Asia1/Shamir/ISR/89 as the previously described VHH M332F, which differs from M658F by only five amino acids. The particle (146S/12S) specificity was further assessed by titration of untreated FMDV authentic particles (UAP) and 12S particles in DAS-ELISAs using only one strain per VHH and calculation of the ratio between the effective concentration (EC) values of 12S/UAP. VHHs were considered to be 146S specific at a ratio > 10, as indicated in Table S2. Based on this criterium, the abovementioned ten serotype A specific VHHs again displayed 146S specificity, as well as the Asia1 binding VHHs M332F and M658F. M651F also displayed some 146S specificity, but with a ratio of 9, did not fulfill this strict criterium.

Among the 146S-specific VHHs, M691F from the green group displayed the broadest strain recognition while showing high 146S specificity. This VHH binds to all 14 tested serotype A strains, except FMDV strain AF72, and did not react with 12S from any of these strains. The other VHH from the green group, M703F, recognized 6 out of 15 serotype A strains in a 146S specific manner. It especially did not bind A/Africa topotype strains. The four VHHs from the purple CDR3 group similarly bound to most of the A/Asia topotype strains but, in addition, bound one A/Africa topotype strain. They did not bind A/Euro SA topotype strains except M652F, which bound A24/Cruzeiro/BRA/55. Of note, strain AF72, which was not recognized by M691F, could be recognized by M659F. Only VHHs M659F and M702F from the purple group showed a consistent 146S-specific binding to all strains recognized. M643F and M652F from the purple group, in general, showed much lower 146S specificity for most strains but were shown to be 146S-specific for strain A/GDMM/CHA/2013. 

Surprisingly, two VHHs exhibited both 146S specificity and cross serotype reactivity. M652F was shown to cross-react with C1/Detmold/FRG/60 with a high 146S specificity. Similarly, M661F, which did not show considerable specificity for 146S particles of the 15 tested serotype A strains, displayed 146S specificity to Asia1/Shamir/ISR/89. The additional color-coded 146S-specific VHHs were found to be highly strain-specific. Among these, VHHs of the orange, gray, and magenta groups bound two strains, A/TUR/14/98 and A/IRN/2/87, whereas the red group VHHs only bound A/TUR/14/98. M651F also showed some 146S specificity for some serotype A strains. 

The remaining 11 novel VHHs were not 146S specific and sometimes even specific for 12S of certain strains. Among them, five VHHs were serotype/strain-specific without 146S specificity. M655F, M662F, and M679F bound several (2–3) serotype A strains, and M675 bound to most of the serotype A strains. In addition, M685F bound to both 146S and 12S of Asia1/Shamir/ISR/89. However, the other six VHHs, M684F, M642F, M665F, M664F, M663F, M680F, reacted broadly with various strains representing several (3–4) serotypes. Especially M663F and M680F bound to all strains analyzed, similar to M3F, and displayed 12S specificity for some strains. 

**Figure 3:**
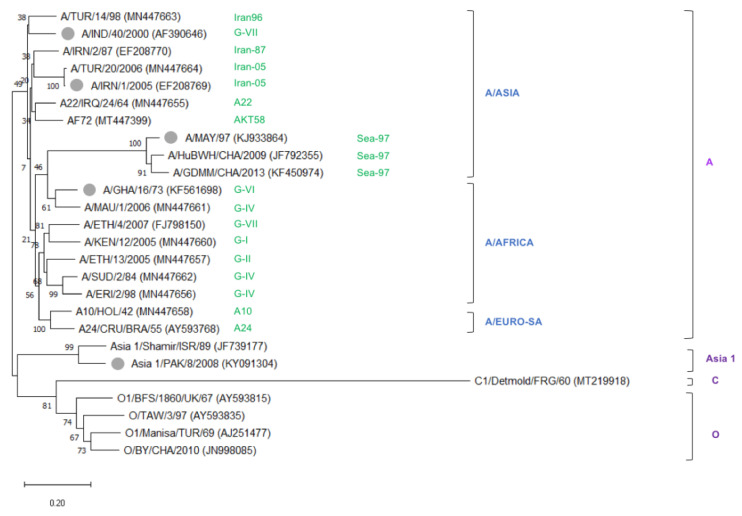
Unrooted neighbor-joining tree showing the phylogenetic relationships between 26 FMDV serotype A, O, C, and Asia 1 strains. In total, 21 strains were assayed for VHH binding by DAS-ELISA, whereas five additional vaccine strains (gray dots) were added to guarantee the tree covers the most important lineages of vaccine strains according to the reports of OIE and FAO [32,33]. VP1 nucleotide sequences were used to generate the tree, with database accession numbers indicated after strain names. The evolutionary distances were computed using the Kimura-2 parameter model, and 500 replicates for bootstrap in MEGA-X. Colors indicate the lineage of the serotype A strain (green), topotype (blue), and serotype (purple).

In summary, the set of 10 VHHs that showed high 146S specificity comprised several VHHs with limited strain recognition but also included four VHHs from two CDR3 groups with much broader strain recognition. Together, this set of VHHs covers all 15 serotype A strains analyzed. Although an additional five serotype A vaccine strains were not used in ELISA, they showed a close phylogenetic relationship to other serotype A strains that were tested (Figure 3). It suggests they could also be recognized by some of these VHHs. Notably, two novel isolated VHHs even showed a cross serotype 146S specificity. 

### 3.3. VHH Specificity for FMDV Particles Fractionated by SDG

**Figure 4:**
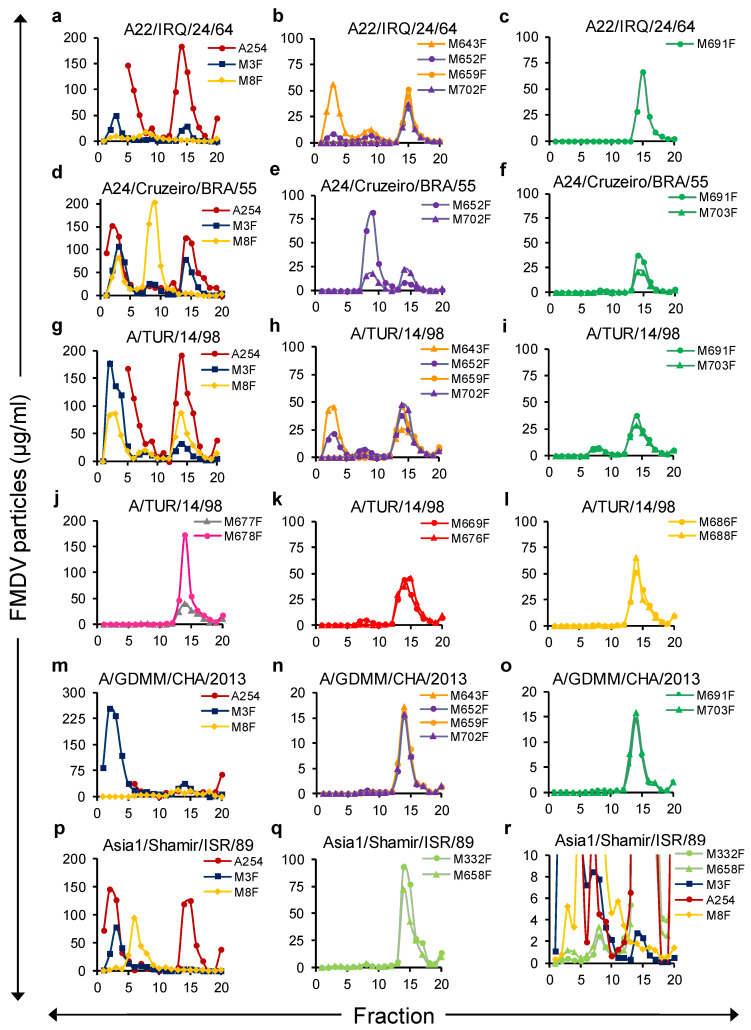
Specificity of novel VHHs for FMDV particles fractionated by SDG. SDG fractions were analyzed using various VHH based DAS-ELISAs. FMDV strains A22/IRQ/24/64 (**a**–**c**), A24/Cruzeiro/BRA/55 (**d**–**f**), A/TUR/14/98 (**g**–**l**), A/GDMM/CHA/2013 (**m**–**o**) and Asia1/Shamir/ISR/89 (**p**–**r**) were used. SDG were layered with a 1:1 mixture of FMDV particle preparations that were either untreated or heated for 1 h at 56 °C to ensure the presence of both 146S and 12S. The 20 fractions were analyzed by the measurement of absorbance at 254 nm to identify the 146S peak and by two ELISAs using the broadly reactive VHHs M3F and M8F to identify all peaks (**a**,**d**,**g**,**m**,**p**). The other panels contain the putative 146S-specific VHHs, with each panel containing clones from a single CDR3 group, except panel (**j**), which contains two VHHs from different CDR3 groups. Clones from the same CDR3 group have identical line colors except for the four VHHs of the same CDR3 group in panels (**b**,**h**,**n)** that were given two colors to allow discrimination of curves. Panel (**r**) represents the same data as panels (**p**,**q**) with a different *Y*-axis scale to enable visualization of lower peaks. Only for the M3F ELISAs, SDG fraction samples and standards were heated for 1 h at 56 °C prior to ELISA. The 12S peak appeared in fractions 1–5, while that of 75S appeared in fractions 7–10 and 146S in fractions 13–16.

To further investigate the absolute particle specificity of the novel isolated VHHs, 146S, 75S, and 12S, particles of four serotype A strains and one Asia1 strain were separated by SDG. The number of FMDV particles in 20 fractions collected from top to bottom was quantified by DAS-ELISA with the novel isolated VHHs as well as the control VHHs M8F and M3F, using standards of crude antigens that were either heated (M3F, 12S specific) or untreated (rest of the ELISAs). The presence of 12S and 75S in the five crude antigens was ignored when using them as standards in this ELISA, which can explain the differences in the FMDV particle concentrations determined by ELISAs or A254. All FMDV particle preparations contained a clear 12S peak in fractions 1–5 as detected by M3F ELISA and 146S peak in fractions 13–16 as detected by A254 (Figure 4a,d,g,m,p). Furthermore, a peak in fractions 7–10 was present for most strains, although very low in the case of A/GDMM/CHA/2013. It represented 75S particles since it was not detected by A254 but was very high when analyzed by M8F ELISA in the case of strain A24/Cruzeiro/BRA/55 (Figure 4d), which is known to produce a high amount of 75S. 

In general, all 13 novel 146S-specific VHHs exhibited a particle specificity consistent with the results reported above. VHHs from the green, red, orange, gray, and magenta groups and M659F, M702F from the purple group did not react with 12S of any of these strains (Figure 4c,f,i–l,o), whereas M643F and M652F from the purple group could detect 12S particles of some but not all four serotype A strains (Figure 4b,e,h,n). M332F and M658F demonstrated only a slight binding to 12S particles of Asia1/Shamir/ISR/89 that could only be observed using an extended *Y*-axis (Figure 4r) and was much lower than the 12S peak detected by M3F ELISA (Figure 4p).

Not all VHHs that display 146S specificity for a strain were able to bind the 75S particle of the same strain. The serotype A binding VHHs showed a complex particle reactivity pattern. Among the VHHs from the purple group, M643F and M652F reacted especially well with the 75S of all four strains, whereas 146S-specific VHHs M659F and M702F showed lower reactivity to 75S and could not detect 75S of A22/IRQ/24/64 (Figure 4b,e,h,n). VHHs from the green CDR3 group similarly could detect 75S of A/TUR/14/98 well (Figure 4i) but reacted very weakly with 75S of A24/Cruzeiro/BRA/55 (Figure 4f) or 75S of A/GDMM/CHA/2013 (Figure 4o) and could not detect 75S of A22/IRQ/24/64 (Figure 4c). Remarkably, the control VHH M8F showed the opposite behavior by reacting well with 75S but not with 146S of strains A22/IRQ/24/64 and A24/Cruzeiro/BRA/55 (Figure 4a, d). For Asia1/Shamir/ISR/89, a small but sharp 75S peak could be seen using both 146S-specific VHHs M332F and M658F (Figure 4r). M8F again showed the opposite and reacted well with 75S but very poorly with 146S. We thus confirmed the absence of 12S binding by 146S-specific VHHs but found remarkable differences in the antigenicity of 146S and 75S particles using 146S-specific VHHs as well as M8F in case of some but not all FMDV strains.

### 3.4. Utilization of Selected VHHs for VLP/Authentic Particle Quantification in ELISAs

**Figure 5:**
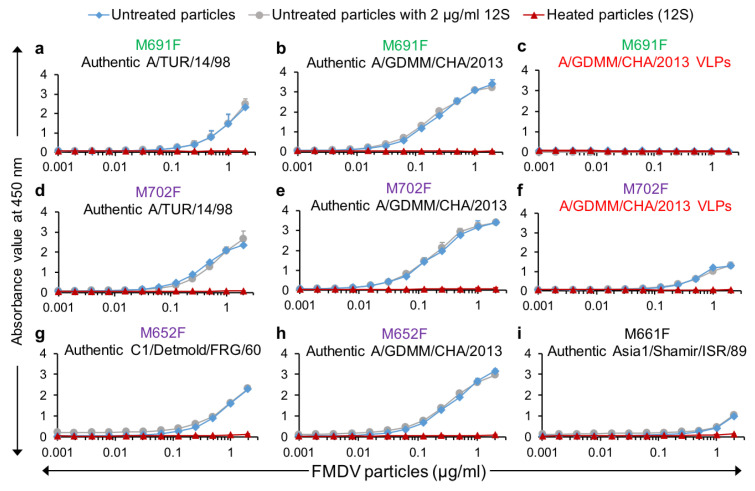
Capsid specificity of selected VHHs and interference of 12S particles in capsid quantification in DAS-ELISAs. Titrations of untreated authentic particles (**a**,**b**,**d**,**e**,**g**–**i**) or untreated VLPs (**c**,**f**) and their 12S particles from four FMDV strains in selected DAS-ELISAs as indicated on top of each panel (VLPs are marked in red). Another titration was done with a 2-fold dilution series of untreated FMDV particles to which a constant amount of 2 µg/mL 12S particles was added to measure interference of 12S particles. VHHs that bind specifically to 146S of serotype A strains are colored green or purple by their CDR3 group. Mean and standard deviation of triplicate ELISAs are given.

VHHs M691F and M702F were further used in DAS-ELISA to measure dilution series of authentic A/TUR/14/98 and A/GDMM/CHA/2013 capsids as well as *E. coli*-produced A/GDMM/CHA/2013 VLPs. Both VHHs again showed high specificity for authentic capsids of both A/TUR/14/98 (Figure 5a,d) and A/GDMM/CHA/2013 (Figure 5b,e). Furthermore, consistent with earlier observed binding of these VHHs to 75S and 146S particles, only M702F but not M691F could recognize A/GDMM/CHA/2013 VLPs (Figure 5c,f). We earlier observed that M652F had low specificity for intact capsids for most FMDV strains with the exception of strain A/GDMM/CHA/2013 and strain C1/Detmold/FRG/60 without titration of FMDV particles (Figure 2). Titration in ELISA confirmed the high capsid specificity of M652F for these two strains (Figure 5g,h). We similarly confirmed the high capsid specificity of M661F for Asia1/Shamir/ISR/89 (Figure 5i). However, both M652F and M661F showed some residual 12S binding (Figure 5g–i), which was not observed for M691F and M702F (Figure 5a–f).

Furthermore, the presence of a constant amount of 12S particles (2 µg/mL) did not interfere with intact capsid quantification by M691F or M702F, whereas M652F and M661F were affected by such spiking with 12S, but only at lower intact capsid concentrations (Figure 5), consistent with their lower capsid specificity. All these ELISAs were done in triplicate for both capsids and 12S particles to further determine the sensitivity of these ELISAs by calculation of the limit of detection (LOD). Both LOD values and 146S specificity are summarized in Table 3. The LOD of A/GDMM/CHA/2013 in M691F and M702F ELISA was lower than those of A/TUR/14/98. The LOD in different ELISAs was thus affected by the FMDV strains used.

### 3.5. Analysis of VHH Binding to Wild-Type and Stabilized Mutant VLPs of Various Serotypes

**Figure 6:**
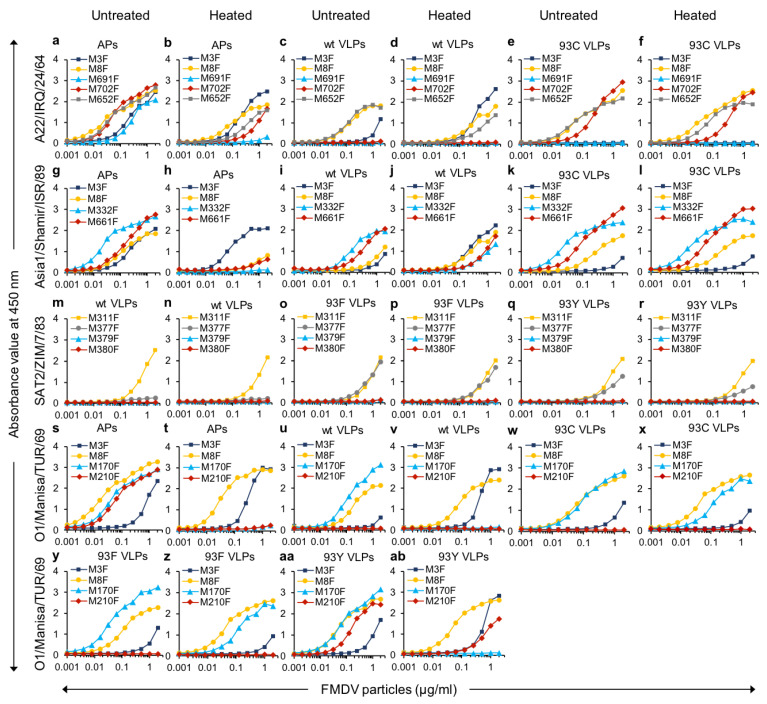
VHH binding to wild-type and stabilized mutant VLPs of various serotypes. The authentic particles (APs) or the different types of VLPs were either untreated or heated at 56 °C for 1 h as indicated on top of each column of panels. The strain used is indicated on the left of each row of panels. Different particles were two-fold titrated in DAS-ELISAs with a 2 µg/mL starting concentration. The control VHHs M8F and M311F, which bound all types of particles, are shown by a yellow curve in each panel. The 12S specific control VHH M3F is shown by a dark blue curve.

After demonstrating antigenic differences between 75S and 146S particles, we further explored the capsid specificity of VHHs using vaccinia virus-produced wt and stabilized mutant VLPs from four different FMDV serotypes. We also used 146S-specific VHHs binding serotypes O and SAT2 strains and some additional control VHHs recognizing both 12S and 146S (Table 1). We used VLPs of strains O1/Manisa/TUR/69, A22/IRQ/24/64 and Asia1/Shamir/ISR/89 since the 146S authentic capsids can be specifically detected by our selected VHHs. However, the SAT2 VLPs were derived from a different strain (SAT2/ZIM/7/83) than used earlier for VHH isolation (SAT2/SAU/2/2000). The binding capabilities of VLPs and the capsid specificities were measured by DAS-ELISAs, shown in Figure 6. The control VHHs, M8F and M311F, show a similar binding curve for VLPs or authentic capsids confirming the correct state of the VLPs. Except for SAT2 VLPs, which were not recognized by M3F, the M3F ELISA also confirmed this and additionally showed that all wt VLPs are heat-labile by their increased binding after heating antigens for 1 h at 56 °C, whereas all mutant VLPs were stabilized.

M691F did not bind both wt- and 93C VLPs of A22/IRQ/24/64 (Figure 6c,e), showing a different antigenicity of VLPs and 146S, similar to the earlier described different antigenicity of 75S and 146S. Furthermore, M702F bound 93C- but not wt VLPs, showing a different antigenicity between two VLPs from the same strain that only differs by a single amino acid mutation. Furthermore, M652F showed much higher binding to wt VLPs than M702F (Figure 6c), in accordance with the earlier observed higher binding of M652F to A22/IRQ/24/64 75S than M702F (Figure 4e). M332F and M661F each bound both wt- and 93C VLPs of Asia1/Shamir/ISR/89 (Figure 6i,k) consistent with the earlier described binding of M332F to 75S. M377F bound to SAT2/ZIM/7/83 93F- and 93Y- but not to wt VLPs (Figure 6m,o,q), again showing a difference in antigenicity using the capsid-specific VHH. M170F binds all four types of (untreated) O1/Manisa/TUR/69 VLPs analyzed, wt, 93C, 93F, and 93Y, whereas M210F only bound 93Y VLPs (Figure 6u,w,y,aa), again showing a different antigenicity of two related VLPs. Interestingly, heating abrogates M170F binding and simultaneously increased 12S-specific VHH M3F binding to 93Y VLPs but not to 93F or 93C VLPs (Figure 6x,z,ab). This suggests a lower heat resistance of O1/Manisa/TUR/69 93Y VLPs as compared to 93F and 93C VLPs. These data, summarized in Table 4, show that some capsid-specific VHHs reveal antigenic differences between wt VLPs and either stabilized VLPs or 146S of serotype A, O, and SAT2. For serotype O, antigenic differences between 93Y- and either 93F or 93C VLPs could also be observed using M210F.

## 4. Discussion

We here report the isolation and characterization of 26 novel VHHs, 13 of which display capsid specificity. We earlier [9,24] referred to such VHHs as ‘146S specific’, but considering the ability of many such VHHs to bind 75S particles and VLPs (see also below), we prefer ‘capsid specific’ to indicate VHHs that do not or inefficiently recognize 12S particles while being able to bind (intact) capsids. The currently available capsid-specific VHHs M170F and M210F do not bind strain O/TAW/3/97 [23] nor O/BY/CHA/2010 (Figure 2). Here we could not isolate novel capsid-specific VHHs using strain O/TAW/3/97, which could be due to the low stability of strains from the serotype O lineage circulating in South East Asia [10]. The novel serotype Asia 1 capsid-specific VHH M658F had high sequence homology to the earlier isolated M332F [24]. Therefore, we focused on the more novel and broader panel of 12 VHHs specific for capsids of serotype A. In total, six of these capsid-specific VHHs showed the typical high specificity for only 1 or 2 out of 15 serotype A strains analyzed, similar to earlier isolated FMDV capsid-specific mAbs [20,21] or VHHs [9,24]. However, the further six VHHs, comprising two CDR3 groups, showed a much broader strain recognition. Four of these (M659F, M702F, M691F, and M703F) also showed a high capsid specificity for all strains analyzed. Together, these VHHs recognized the 15 serotype A strains analyzed that cover the most important 14 prophylactic vaccine genotypes/lineages [32,33]. This is surprising since serotype A is genetically the most variable among the seven FMDV serotypes [25,26]. It suggests that isolation of capsid-specific VHHs with broad strain recognition is also feasible for other serotypes. The VHHs M661F and M652F even showed capsid specificity for strains from two different serotypes but also showed some residual 12S binding that varied between strains, making them less suitable for quantification of FMDV capsids in the vaccine. Therefore, primarily M702F and M691F from two different CDR3 groups were further evaluated for quantification of FMDV capsids. The two DAS-ELISAs using these VHHs were shown to have a high capsid specificity even when spiking the samples with 2 µg/mL 12S particles. The ELISAs also enabled broad strain recognition and had a low LOD using prototype strains A/TUR/14/98 (41–97 ng/mL) or A/GDMM/CHA/2013 (17–21 ng/mL). This compares favorably with the LOD of other methods for FMDV antigen quantification, that was reported to be 0.6 µg/mL for HPLC [14] and in the range of 0.3–1 µg/mL for SDG [13,29]. 

Most previously published DAS-ELISAs for quality control of conventional vaccines were used to detect 146S particles but not 75S or VLPs [9,17,18,19,20,21,24,34]. One study used SDG fractionation and ELISA analysis with mAbs that react with all FMDV particles to detect 75S, although not in a quantitative or high-throughput manner [35]. We, therefore, studied the recognition of 75S particles, as well as VLPs produced by either *E. coli* or vaccinia virus by the novel capsid-specific VHHs as well as earlier isolated ones, which is summarized in Table 4. Unexpectedly, in the case of both strains A24/Cruzeiro/BRA/55 and A22/IRQ/24/64, M691F reacted much less efficiently with 75S than 146S particles, while the control VHH M8F, which also bound 12S particles, showed the opposite specificity by reacting more weakly with 146S than 75S particles. Interestingly, such a difference in antigenicity was not observed with strains A/TUR/14/98 and A/GDMM/CHA/2013. Earlier studies did not reveal differences in antigenicity between 146S and 75S using various mAbs [7,36], presumably because these mAbs were not capsid-specific. The ability of capsid-specific VHHs to bind 75S particles was generally consistent with the analysis of VLPs. The VHHs M702F and especially M652F, which belonged to the same CDR3 group, reacted more efficiently with 75S particles and VLPs than M691F. We also showed that the earlier isolated VHHs M170F, M332F, and M377F can be used for quantification of multiple VLPs of serotypes O, Asia 1, and SAT2, respectively. These findings underscore the value of VHHs in the quality control of VLP vaccines. 

Vaccinia virus-produced VLPs of serotypes O, A, Asia 1, and SAT2 comprised both wt VLPs as well as corresponding particles with an amino acid substitution of VP2 residue 93 to F, Y, or C resulting in the stabilization of VLPs against dissociation into 12S particles upon heating for 2 h at 56 °C, although the 93Y VLP of O1/Manisa/TUR/69 showed only partial stabilization [3,5]. Consistent with such stabilization, we observed that heating of wt VLPs but not of stabilized mutants resulted in increased ELISA signals using the 12S-specific M3F and decreased ELISA signals with the various capsid-specific VHHs, while the 93Y VLP of O1/Manisa/TUR/69 showed only partial stabilization. VP2 amino acid 93 is located close to the 2-fold axis where two pentamers associate, which explains the stabilizing effect of specific amino acid substitutions at this position [3,5,37]. The wt VLPs of serotype O, A, and SAT2 also showed strongly reduced reactivity with the capsid-specific VHHs M210F, M702F, and M377F, respectively, although they could recognize at least one stabilized VLP of each serotype. Thus, despite their higher sequence homology, the wt VLPs showed a different antigenicity from 146S particles that was not observed with some stabilized VLPs. Such differences in antigenicity due to VLP stabilization have not been observed before. However, the introduction of the VP2 mutation 93Y into SAT2 virions rather than VLPs resulted in altered antigenicity using mAb GD12 [37]. These authors speculate that mAb GD12 recognizes a site that is altered due to VP2 mutation 93Y. However, this cannot explain the observation that the capsid-specific VHHs M702F, M210F, and M377F reacted with 146S particles while not reacting with wt VLPs, that have the same residue at VP2 position 93. Similarly, the different antigenicity of 75S and 146S particles as revealed by M691F and M8F binding is difficult to explain by specific amino acid mutations, as both particles are derived from the same polyprotein. The presence of uncleaved VP0 (VP4 + VP2) in 75S but not in 146S particles is also unlikely to cause the difference in antigenicity since uncleaved VP0 is only a minority population in 75S particles [38]. Thus, differences in VLP primary sequence do not appear to explain the differences in antigenicity observed using capsid-specific VHHs.

Recently, the Cryo-EM structure of the capsid-specific VHH M170F with 146S particles was elucidated [39]. M170F was found to bind the VP3 GH loop that forms a different structure in 12S particles, suggesting that conformational differences between 12S and 146S explain the capsid specificity of M170F. We, therefore, hypothesize that, similar to M170F, other VHHs bind specifically to 146S but not 12S by binding to similar conformational sites. Such a hypothesis would also imply that conformational differences between 146S and either natural 75S or recombinant VLP empty capsids cause the observed differences in antigenicity. Such a hypothesis is consistent with the often reported structural plasticity and altered antigenicity of several other picornaviruses, such as poliovirus [40], enterovirus 71 [41], and equine rhinitis A virus [42]. Furthermore, structural differences between 146S and 75S particles of A22/IRQ/22/64 were identified by X-ray crystallography [38], and an altered conformation of the VP3 GH-loop was shown to affect the antigenicity of an A22/IRQ/22/64 mutant virus [43]. Finally, X-ray crystal structures of wt and 93C VLPs of A22/IRQ/24/64 showed an altered conformation of the VP3 GH-loop as compared to authentic virus where the folding of this loop of 93C VLPs was more similar to the authentic A1061 virus than wt VLPs [5,44]. However, structural differences were not observed between wt and 93Y VLPs of serotype SAT2 [3]. Taken together, this suggests that the capsid-specificity, in general, relies on the recognition of different conformations of antigenic sites.

Irrespective of the molecular basis of differences in antigenicity, which is one of the focus points of our current research, our capsid-specific VHHs prove useful tools not only for VLP quantification but also for qualitative assessment of their suitability for vaccine use. In this respect, the demonstration that several stabilized mutant VLPs of serotypes A, O, and SAT2 [3] show more similar antigenicity to 146S than wt VLPs supports their use in vaccines. The similarities in antigenicity between 146S and 93Y VLP of O/Manisa//TUR/69 in contrast to the 93F or 93C VLPs would favor the use of the 93Y VLPs despite the slightly lower thermostability. On the other hand, freshly prepared wt and stabilized VLPs similar to the ones used in this study produced equivalent titers of neutralizing antibodies [3,5] and levels of protection [5], indicating that stabilization is perhaps less essential. Furthermore, such VLPs and authentic viruses induce similar bovine dendritic cell activation, T cell proliferation, and antibody responses to FMDV [45]. Thus, the relevance of the observed differences in antigenicity for the ability to induce protective immunity still needs to be determined.

## 5. Conclusions

The newly-developed VHHs presented here are promising tools for the quality control of vaccines based on 146S, 75S, or (stabilized) VLPs produced by *E. coli*, vaccinia virus, or baculovirus. In addition to their practical use for vaccine quality control, the VHHs can be used to gain more insight into the structure-function relationship of FMDV capsids.

## Figures and Tables

**Table 1:** Previously isolated VHHs used for phage display selection and ELISA.

VHH	Serotype Specificity	Particle Specificity	Reference
M8F and M220F	O, A, Asia1, C	146S and 12S	Harmsen et al., 2007 [23]
M3F	O, A, Asia1, C	12S	Harmsen et al., 2007 [23]
M23F	O	146S and 12S	Harmsen et al., 2007 [23]
M170F and M210F	O	146S	Harmsen et al., 2007 [23]
M98F	Asia1	146S and 12S	Harmsen et al., 2017 [24]
M332F	Asia1	146S	Harmsen et al., 2017 [24]
M321F and M326F	A	146S and 12S	Harmsen et al., 2007 [23]
M311F	SAT2	146S and 12S	Harmsen et al., 2017 [24]
M377F, M379F, and M380F	SAT2	146S	Harmsen et al., 2017 [24]

**Table 2:** Sequence and phage display selection information of 26 newly isolated VHHs.

VHH ^a^	VHH Subfamily ^b^	Phage Display Selection		CDR3 Sequence	VHHs from Same CDR3 Group (Number of Amino Acid Differences) ^d^
		FMDV Strain ^c^	Capture VHH		
M643	1	A/TUR/14/98	M8F	ATDPSWTAFKNAARYDY	M659(17), M702(14), M652(13)
M652	1	A/TUR/14/98	M8F	ATDQSWTASKNAARYDN	M659(12), M702(13), M643(13)
M659	X	A22/IRQ/24/64	M321F	AADQSWTASMNPARYDY	M702(15), M643(17), M652(12)
M702	1	A22/IRQ/24/64	M321F	AADPSWTASMNAARYDY	M659(15), M643(14), M652(13)
M691	X	A24/Cruzeiro/BRA/55	M326F	KLEDWITGQTY	M703(9)
M703	2	A/TUR/14/98	M8F	NLKDWITGQTY	M691(9)
M686	1	A/TUR/14/98	M8F	AAKYKSTFYTTMDVQYDY	M688(7)
M688	1	A/TUR/14/98	M8F	AAKYQSTFYSTMDVQYDY	M686(7)
M669	X	A/TUR/14/98	M8F	TARGWDDTEYPY	M676(8)
M676	X	A/TUR/14/98	M8F	VGRGWDDTEYPY	M669(8)
M677	1	A/TUR/14/98	M8F	AAGWSGMAPSRANSYAY	NA
M678	2	A/TUR/14/98	M8F	NTVRRVATLSGSSSGS	NA
M651 ^e^	1	A22/IRQ/24/64	M321F	AVEEDSGLNYDAGWYDY	NA
M661	1	A/TUR/14/98	M8F	AADNAVVVPTTTRGYDY	NA
M655 ^e^	1	A24/Cruzeiro/BRA/55	M326F	AARTSPYYSAYASHYDY	NA
M662	1	A24/Cruzeiro/BRA/55	M326F	AASGSYNDFFEDDVDY	NA
M679	1	A/TUR/14/98	M8F	AAREGPAIYNSDRYFPY	NA
M675	1	A22/IRQ/24/64	M321F	AAREDYYYTGHEYDY	NA
M685	1	A22/IRQ/24/64	M8F	AADLDRWESVATMIYDYDY	NA
M658	1	Asia1/Shamir/ISR/89	M98F	AAAWSFRSDYGARIKSAYDF	M332(5)
M684	1	A22/IRQ/24/64	M321F	AVGRSLVRDSRAYDY	NA
M642	2	Asia1/Shamir/ISR/89	M98F	NARATSGWASYVT	NA
M665	2	O/TAW/3/97	M8F	YAGEAAGWGSHVY	NA
M664	2	O/TAW/3/97	M23F	NVRDQAGLGYSDY	NA
M663	1	A24/Cruzeiro/BRA/55	M326F	TAGRSYSSNPAAYNY	NA
M680	1	A24/Cruzeiro/BRA/55	M326F	AADRSLTTRDVAYDY	NA

^a^ The 12 VHHs of the 6 CDR3 groups comprising the 10 VHHs that bind specifically to 146S of serotype A strains are color-coded by their CDR3 group in purple, green, orange, red, gray and magenta (VHHs in black belong each to a unique CDR3 group). ^b^ Subfamilies were earlier defined [31]. X indicates VHHs that could not be classified. ^c^ The underlined strain indicates that 12S from another strain was used as a competitor in panning. ^d^ All clones from different CDR3 groups showed at least 20 amino acid differences, except M665 and M642 that showed 13 amino acid differences. NA, not applicable. ^e^ M651F and M655F are the only two VHHs containing a potential N-glycosylation site. The Asn residue of the potential N-glycosylation site was located at IMGT positions 57 and 84, respectively.

**Table 3:** The limit of detection (LOD) of VHH-based DAS-ELISAs. Various authentic particles (AP), VLPs, or their 12S particles prepared by heating were titrated in ELISA in triplicate.

VHH ^a^	FMDV Strain	Capsid Type	LOD (ng/mL) ^b^		Ratio 12S/Intact Capsid ^d^
			Intact Capsid	12S	
M691F	A/TUR/14/98	AP	97	>2000 ^c^	21
M691F	A/GDMM/CHA/2013	AP	21	>2000	95
M691F	A/GDMM/CHA/2013	VLP	>2000	>2000	NA ^e^
M702F	A/TUR/14/98	AP	41	>2000	49
M702F	A/GDMM/CHA/2013	AP	17	>2000	119
M702F	A/GDMM/CHA/2013	VLP	162	>2000	12
M652F	C1/Detmold/FRG/60	AP	76	>2000	27
M652F	A/GDMM/CHA/2013	AP	23	>2000	86
M661F	Asia1/Shamir/ISR/89	AP	341	>2000	5.9

^a^ VHHs that bind specifically to 146S of serotype A strains are colored green or purple by their CDR3 group. ^b^ The LOD was calculated based on the average absorbance value of background plus three times the standard deviation. ^c^ >2000 indicates LOD not reached at the highest FMDV particle concentration used (2 μg/mL). ^d^ The ratio 12S/Intact capsid (146S specificity) was determined by LOD of 12S/LOD of capsid. ^e^ NA, Not applicable.

**Table 4:** Summary of VHH binding to different FMDV authentic particles and VLPs.

VHH ^a^	Binding to FMDV Particles ^b^							FMDV Particle Specificity ^c^
	146S	75S	wt VLP	93C VLP	93F VLP	93Y VLP	12S	
*A/TUR/14/98*								
M8F	+	+	ND	ND	ND	ND	+	None
M652F	+	+	ND	ND	ND	ND	+	None
M702F	+	+	ND	ND	ND	ND	-	Capsid
M691F	+	+	ND	ND	ND	ND	-	Capsid
*A22/IRQ/24/64*								
M8F	+/-	+	+	+	ND	ND	+	75S preference
M652F	+	+	+	+	ND	ND	+	None
M702F	+	-	-	+	ND	ND	-	Capsid
M691F	+	-	-	-	ND	ND	-	146S
*A24/Cruzeiro/BRA/55*								
M8F	+/-	++	ND	ND	ND	ND	+	75S preference
M652F	+	++	ND	ND	ND	ND	+/- ^d^	75S preference ^d^
M702F	+	+	ND	ND	ND	ND	-	Capsid
M691F	+	+/-	ND	ND	ND	ND	-	Capsid
*A/GDMM/CHA/2013* ^e^								
M8F	+	+	ND	ND	ND	ND	+	None
M652F	+	+	ND	ND	ND	ND	-	Capsid
M702F	+	+	+	ND	ND	ND	-	Capsid
M691F	+	+	-	ND	ND	ND	-	Capsid
*Asia1/Shamir/ISR/89*								
M8F	+/-	++	+	+	ND	ND	+/-	75S preference
M332F	+	+	+	+	ND	ND	-	Capsid
M661F	+	ND	+	+	ND	ND	-	Capsid
*O1/Manisa/TUR/69*								
M8F	+	+	+	+	+	+	+	None
M170F	+	+	+	+	+	+	-	Capsid
M210F	+	ND	-	-	-	+	-	Capsid
*SAT2/SAU/2/2000 (authentic particles) or SAT2/ZIM/7/83 (VLPs)*								
M311F	+	ND	+	ND	+	+	+	None
M377F	+	ND	-	ND	+	+	-	Capsid

^a^ Underlined VHHs showed 146S specificity in earlier published data. VHHs that bind specifically to 146S of serotype A strains are colored green or purple by their CDR3 group. ^b^ The binding behavior of VHHs is defined as follows: + is binding when a peak is visible in SDG or ELISA signal in Figure 6 (absorbance > 0.3 at 2 µg/mL FMDV particles); - is no binding (absorbance < 0.3 at 2 µg/mL FMDV particles); +/- is slight binding (a peak is visible SDG, but not as high as with other VHHs); ND is not determined; ++ indicates better binding than +. ^c^ The particle specificity of VHHs is defined as follows. Capsid: binding both 146S and 75S; underlined Capsid: binding 146S, 75S and VLPs; none: binds all particles; 146S: binds only 146S; 75S preference: binds all particles but with a preference for 75S. ^d^ M652F showed an inconsistency: it showed capsid-specific binding with a preference for 75S when analyzing SDG, but could bind 12S obtained by heating (Figure 2). ^e^ A/GDMM/CHA/2013 wt VLPs are the only VLPs produced in *E. coli*.

## Data Availability

Nucleotide sequences of FMDV P1 coding regions were submitted to the Genbank database under accession numbers MN447655-MN477664.

## Supplementary Materials

The following are available online at https://www.mdpi.com/article/10.3390/vaccines9060620/s1, Figure S1: FMDV intact capsid- and strain-specificity of novel VHHs determined by DAS-ELISA, Table S1: Antigens used for llama immunization, Table S2: Specificity of VHHs for 146S particles after titration in DAS-ELISA using a single FMDV strain.
