# Supplementary material for: Novel Capsid-Specific Single-Domain Antibodies with Broad Foot-and-Mouth Disease Strain Recognition Reveal Differences in Antigenicity of Virions, Empty Capsids, and Virus-Like Particles
Source: Vaccines (Basel). 2021 Jun 8;9(6):620. doi: 10.3390/vaccines9060620 (PMC8227720; doi:10.3390/vaccines9060620)
Supplement: Supplementary file 1 [file vaccines-09-00620-s001.zip › vaccines-1227967-supplementary.pdf]

Supplementary Materials

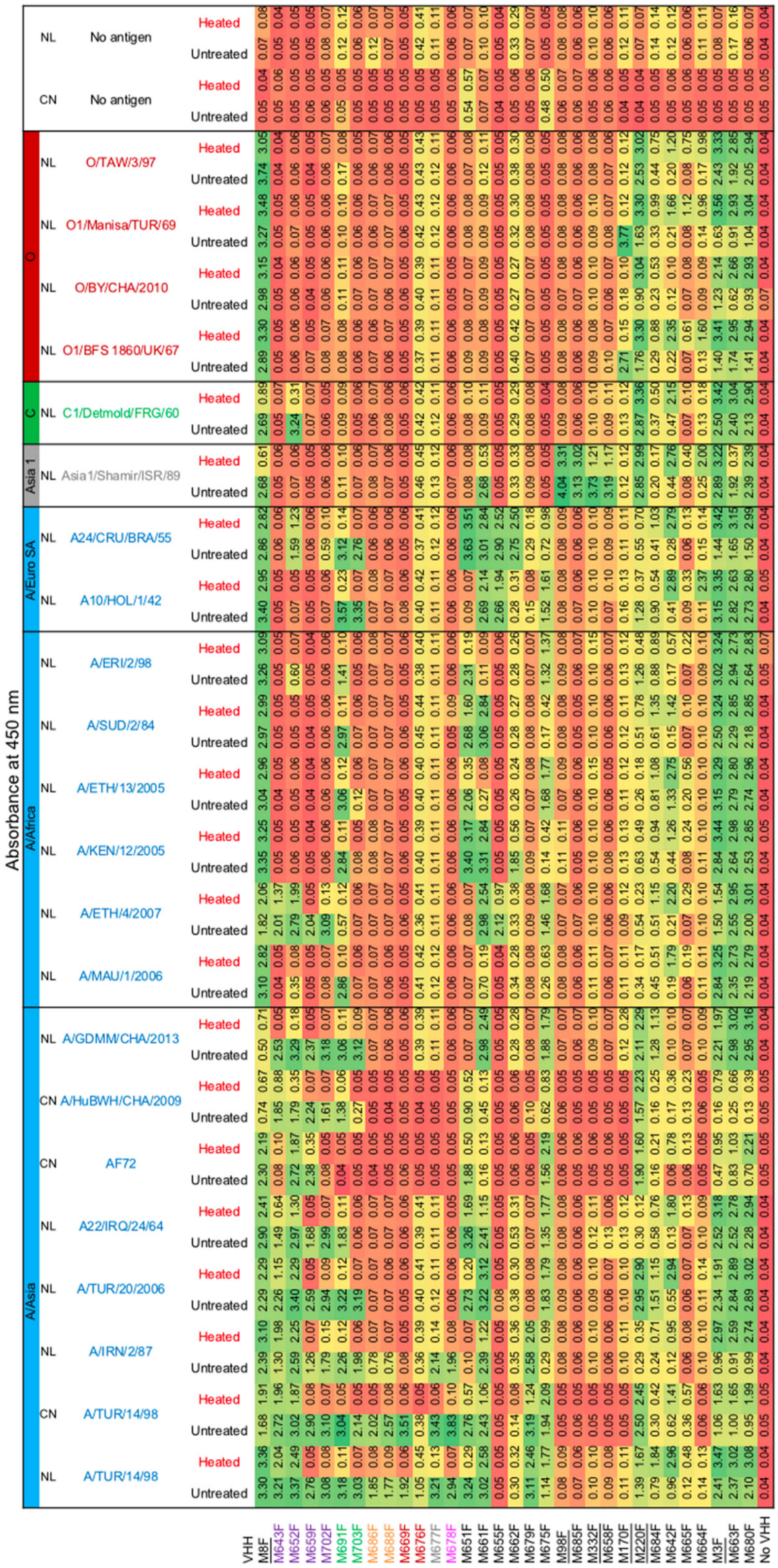

Figure S1. FMDV intact capsid- and strain specificity of novel VHHs determined by DAS-ELISA. Absorbance values at 450 nm are indicated using a red/green coloring scheme. Specificity for intact capsids was determined by binding of 32 VHHs to authentic FMDV particles of 21 strains of different serotypes that were either heated for 1 h at 56 ⁰C (12S) or untreated. A total of 15 FMDV serotype A strains, 4 serotype O strains, one serotype C and one Asia1 strain were used. The 12 VHHs of the 6 CDR3 groups comprising the 10 VHHs that bind specifically to 146S of serotype A strains are color-coded by their CDR3 group. The control VHHs isolated earlier are underlined. The VHHs are arranged from top to bottom according to their CDR3 group and their strain and particle specificity. The FMDV strains are arranged from left to right according to their phylogenetic relationship (Figure 3). ELISAs were performed in the Netherlands (NL) or China (CN), as indicated.

Table S1. Antigens used for llama immunization. Antigens were either PEG precipitated crude antigens or further purified by sucrose density gradient (SDG). One llama was immunized by infection with live virus.

Llama	FMDV Strains	FMDV Antigen Used for Immunization	Reference
3049	A24/Cruzeiro/BRA/55, Asia1/Shamir/ISR/89	SDG purified 146S	
6058	O1/Manisa/TUR/69, Asia1/Shamir/ISR/89, A24/Cruzeiro/BRA/55, A22/IRQ/24/64	Crude	Harmsen et al. 2007 [23]
6666	O1/Manisa/TUR/69, Asia1/Shamir/ISR/89, A24/Cruzeiro/BRA/55, A22/IRQ/24/64	SDG purified 146S [a]	Harmsen et al. 2007 [23]
7212	O1/Manisa/TUR/69	Live virus	Harmsen et al. 2007 [23]
9245	O1/Manisa/TUR/69, A22/IRQ/24/64	SDG purified 146S	This study
9246	A/TUR/14/98	SDG purified 146S	This study

[a] Llama 6666 was also immunized with a synthetic peptide representing the GH-loop of strain O1/Manisa/TUR/69 [23].

Table S2. Specificity of VHHs for 146S particles after titration in DAS-ELISA using a single FMDV strain. Both untreated authentic particles (UAP) or heated particles (12S) were titrated in DAS-ELISA with eight 3-fold dilutions starting at 1 μg/ml. Then the Effective Concentration (EC) to reach an absorbance value at 450 nm of 1 was calculated.

VHH [a]	FMDV Strain	EC (ng/ml)		EC Ratio 12S/UAP [b]
		UAP	12S	
M8F	A/TUR/14/98	26	48	1.9
M643F	A/TUR/14/98	9.3	10	1.1
M652F	A/TUR/14/98	11	13	1.2
M659F	A/TUR/14/98	15	247	16.4
M702F	A/TUR/14/98	3.9	52	13.2
M691F	A/TUR/14/98	19	209	10.9
M703F	A/TUR/14/98	78	>1,000	>12.8
M686F	A/TUR/14/98	7.4	95	12.9
M688F	A/TUR/14/98	14	340	12.9
M669F	A/TUR/14/98	5.7	361	63.3
M676F	A/TUR/14/98	40	>1,000	>24.9
M677F	A/TUR/14/98	26	267	10.4
M678F	A/TUR/14/98	10	277	26.9
M651F	A/TUR/14/98	85	764	9.0
M661F	A/TUR/14/98	14	20	1.4
M655F	A24/Cruzeiro/BRA/55	22	31	1.4
M662F	A/TUR/14/98	537	182	0.3
M679F	A/TUR/14/98	21	44	2.0
M675F	A/TUR/14/98	46	6	0.1
M98F	Asia1/Shamir/ISR/89	7.5	36	4.8
M685F	Asia1/Shamir/ISR/89	27	55	2.0
M332F	Asia1/Shamir/ISR/89	5.2	418	80.4
M658F	Asia1/Shamir/ISR/89	4.5	334	74.3
M684F	A/TUR/14/98	161	24	0.1
M642F	A/TUR/14/98	147	16	0.1
M665F	A/TUR/14/98	>1,000	437	<0.4
M664F	Asia1/Shamir/ISR/89	367	72	0.2
M3F	A/TUR/14/98	249	36	0.1
M663F	A/TUR/14/98	193	31	0.2
M680F	A/TUR/14/98	198	103	0.5

[a] Underlined VHHs were earlier published [23,24]. The 12 VHHs of the 6 CDR3 groups comprising the 10 VHHs that bind specifically to 146S of serotype A strains are color-coded by their CDR3 group. [b] Underlined EC ratio 12S/UAP>10 is considered 146S specific.
